# The National Dental Association

**Published:** 1879-08

**Authors:** 


### ARTICLE II.

### *The National Dental Association.*

The Eleventh Annual Session of the National Dental Association was held in Augusta, Georgia, commencing July 8th, 1879, and proved to be one of the largest and most beneficial sessions of the dental profession ever held in the Southern States.

An address of welcome on the part of the City of Augusta, was delivered by Dr. Geo. H. Winkler, in the following happy manner:

He thought they would have a satisfactory and creditable meeting, for this Association honored Georgia by meeting within her borders and deliberating in Augusta. He congratulated the Association upon its prominence, which he trusted would be second to none at an early day, and for the high standing of the profession in the States and in the country. It was proposed to take the delegates up the canal and let them float upon the bosom of Lake Olmstead in those beautiful steamers, one of which sunk last week with a party of negro excursionists. (Laughter.) The steamer, however, floats once more and is all right again. We then, he said, propose to show you all the dam bulkhead—or bulkhead dam—a work of so much engineering skill. We propose then gentlemen, continued the facetious orator, to show you one Uriah, not the Biblical character and friend of David, but Lexius Henson's steward, so adept in building lemonades and making whisky punches. (Laughter.) The President wisely informs me that being a temperance reformer, and having only joined a lodge last night, I should not mention these things; but I do mention them because I know they will make you all feel good. (Laughter.) And then gentlemen, when we return to town from this excursion, to show that there is nothing mean about us, we propose to make you pay for the fun. (Laughter.) But gentlemen, I do welcome you to the city, and you know I do. (Applause.)

Dr. J. D. McKeller, of Reynolds, Taylor county, Ga., was then introduced by President Wardlaw, of the Georgia Society, and in behalf of the State Society welcomed the National Association. He hoped that the dignity, importance and honor of the profession would be upheld. The Association would develop opposition to pet schemes and hobbies, but every one should receive defeat coolly and well, for opposition is the law of progress. He welcomed the National Association to Georgia as friends, as brothers, and as laborers in a common cause.

The National Dental Association was then convened by the President, Prof. F. J. S. Gorgas, of Baltimore, Md., after which the roll of members was called by Dr. E. S. Chisholm, of Ala., Recording Secretary, the following officers responding and a large number of members:

*President.*—Dr. F. J. S. Gorgas.

1*st Vice President.*—Dr. L. D. Carpenter.

2*nd Vice President.*—Dr. J. R. Walker.

*Recording Secretary.*—Dr. E. S. Chisholm.

*Treasurer.*—Dr. H. A. Lowrance.

*Members.*—Drs. Ervin Floyd, Fayetteville, N. C.; Vines E. Turner, Raleigh, N. C.; D. L. Boozer, Columbia, S. C.; J. Quattlebaum, Blythewood, S. C.; G. H. Winkler, Augusta, Ga.; S. Hape, Atlanta, Ga.; W. R. Bull, Charleston, S. C.; F. J. S. Gorgas, Baltimore, Md.; J. S. Thompson, Greenville, S. C.; J. D. McKeller, Reynolds, Ga.; W. C. Wardlaw, Augusta, Ga.; J. A. Herman, Nashville, Tenn.; J. H. E. Millhouse, Blackville, Ga.; B. J. Quattlebaum, Williston, S. C.; R. W. Thornton, Calhoun, Ga.; R. C. Roberts, Allendale, Ga.; J. A. Harman, Prosperity, Ga.; J. P. Holmes, Macon, Ga.; G. W. McElhany, West Point, Ga.; J. H. Coyle, Thomasville, Ga.; E. Parsons, Savannah, Ga.; L. D. Carpenter, Atlanta, Ga.; D. P. Holloway, Americus, Ga.; W. F. Tignor, Columbus, Ga.; T. T. Moore, Columbia, S. C.; J. R. Thompson, Newberry, S. C.; J. B. Patrick, Charleston, S. C.; A. G. Bouton, Savannah, Ga.; T. P. Legare, Camden, S. C.; R. B. Adair, Gainesville, Ga.; C. C. Patrick, Charleston, S. C.; E. S. Chisholm, Tuscaloosa, Ala.; T. M. Allen, Eufaula, Ala.; H. J. Mozon, Blackville, Ga.; M. A. Bland, Charlotte, N. C.; B. H. Teague, Aiken, S. C.; G. F. S. Wright, Columbia, S. C.; J. P. H. Brown, Augusta, Ga.; S. B. Barfield, Macon, Ga.; W. W. Ford, Macon, Ga.; G. B. White, Chester, S. C.; J. M. Mason, Columbus, Ga.; N. C. Quillan, Thomson, Ga.; Geo. W. H. Whitaker, Sandersville, Ga.; J. R. Walker, New Orleans, La.; D. L. Wilson, Abbeville, S. C.; H. Parker, Edgefield, S. C.; D. E. Everett, Raleigh, North Carolina; R. M. Gage, Nashville, Tenn.

Many others arrived after the morning session whose names will appear in the volume of proceedings.

The Presidents of the Georgia, South Carolina and North Carolina Dental Societies were invited to seats on the stage.

The names of Drs. Patrick, Parsons and McElhany were then announced as the Committee on Membership.

The minutes of meeting at Niagara Falls, August, 1878, were read and approved. President Gorgas then delivered the annual address. He requested the co-operation of all to the high development and progressive advancement of dental surgery and dental literature. People are often imposed upon, and confidence often given to men unworthy of it. It is our duty so to educate the public as to enable them to bring common business sense in selecting a dentist. Our profession will compare most favorably with all others, for its foundation is science, and its aims the good of mankind. Dr. Gorgas then dwelt upon the physiological structure of the teeth, and its wonderful functions connecting the past with the present, uniting the mammals of geology to the human beings of to-day. Dr. Gorgas advocated an improvement in preliminary education. The distinguished speaker then dwelt upon talent and genius and the tendency of science. Speculative thought may be apparently at variance with divine revelation, but the demonstrative science must always prove a single and inspired origin in the vegetable and organic kingdom. Dr. Gorgas concluded that all life must have originated from a personal Creator. The President hoped that this year a union would be made between the American Dental Associations and State Organizations, to form a general National Association. He deprecated the fallacy of sinking the dental with medical science, and said that the combination was not necessary, and that a dentist need not have received a medical education. He cautioned all laborers in the science not to allow the ambitious soul to struggle at the expense of the material body. He feelingly alluded to the death of prominent and distinguished workers in the profession, among the number the late Profs. P. H. Austen, of Baltimore, and John H. McQuillen, of Philadelphia.

After the address the regular business of the session was commenced. The name of the Association was changed from the Southern Dental Association to the National Dental Association.

The following order was determined upon for discussing important subjects:

1. Dental Education. 2. Dental Histology and Microscopy. 3 Causes of Deterioration of Teeth. 4. Care of the Teeth during Gestation and Lactation. 5. Deciduous Teeth —their relation to the Permanent and their Treatment. 6. Transplanting and Replanting Teeth. 7. Relative Merits of Cohesive and Non-cohesive Gold as a Filling for Teeth. 8. Contour Fillings. 9. Conservative Treatment of Dental Pulp. Dead Teeth and Alveolar Abscess. 10. Relative Merits of Rubber and Celluloid. 11. The use of Plastic Materials for Filling Teeth.

It was determined that the daily sessions be from 9:30 A. M. to 1:30 P. M.; 3:30 to 6:30 P. M. and 9 to 11 P. M., and that Clinics be held daily from 8:30 to 10:30 A. M.

The subject of "Dental Education" was taken up and discussed by Drs. Wright, of South Carolina, Mason, of Macon, Walker, of New Orleans, and others. The next regular subject "Dental Physiology and Surgery" was introduced and discussed by Drs. John H. Coyle, McKeller, Patrick, Holmes, Carpenter and Tigner.

### SECOND DAY'S PROCEEDINGS.

The Association was called to order at 10:30 A. M. by the President, Prof. Gorgas. Dr. E. Parsons, of Savannah, Ga., read an interesting paper on "Dental Physiology."

The following committee was appointed to report on Mechanical Appliances: J. R. Walker, of New Orleans; G. F. S. Wright, of Columbia; and V. E. Turner, of Raleigh.

Dr. Winkler extended, on behalf of the dentists of Augusta, an invitation to go up the canal this afternoon on a pleasure trip, which was unanimously accepted.

The following committee on publications was appointed: Drs. E. S. Chisholm, H. A. Lowrance and W. W. Ford.

The Executive Committee consisting of Drs. W. C. Wardlaw, Augusta, Ga.; G. H. Winkler, Augusta, Ga.; G. W. H. Whitaker, Sandersville, Ga., then made a report which was accepted.

Some very able papers were then read on Dental Physiology and fully discussed, a large number of the members participating.

The hour of adjournment having arrived, it was determined to devote the afternoon to the excursion to the Locks, which had been tendered by the dentists of Augusta. The members accordingly went up the canal on Commodore Armstrong's fleet, and had a delightful time.

The refreshments were provided by Mr. Hugh Boyle, and were served in fine style. On the return trip a number of the party were called on and made impromptu speeches.

At the evening session interesting papers on Histology and Microscopy by Drs. W. H. Atkinson, of New York, and S. P. Cutler, of Memphis, Tenn., were read.

### THIRD DAY'S PROCEEDINGS.

After the Morning Clinics, the Association was called to order by the President, Prof. Gorgas, and the subject of Dental Physiology continued.

Dr. J. P. Holmes, of Macon, read a paper on Dental Chemistry, which was discussed by Drs. Winkler, Ford, Moore, Walker, Brown, McKeller, Teague and Parsons.

The subject of Operative Dentistry was then taken up and interesting discussions followed. Also, an interesting exhibit of appliances for fracture of the Lower Alveolar Border by Dr. Harker.

At the opening of this morning's session an interesting essay on Dental Therapeutics was delivered by Dr. G. S. Wright.

Dr. T. T. Moore discussed a case of Irregularity, exhibiting models, casts, &c.

Dr. E. S. Chisholm read a highly interesting paper on the Diagnosis and Treatment of Nerve Exposure.

Dr. Parker exhibited specimens of peculiar and anomalous teeth.

Dr. J. M. Mason, of Columbus, was elected a member of the National Association.

The Association was treated to a most interesting, eloquent and instructive address by Dr. J. B. Patrick, of South Carolina.

In discussing Operative Dentistry, Dr. Teague thought the theory of the "New Departure" much better than the practice.

Dr. Carpenter highly recommended and thought stannous gold made the best plastic filling. Dr. Patrick also favored the stannous gold, while Dr. Winkler said that the best amalgam he had ever used was made by Dr. Harker.

Drs. Parker and Patrick gave interesting cases in practice, while Ludwig's Cement, White's Agate Cement, Justi's Cement and Fletcher's Porcelain and other plastic materials, were also discussed.

The morning session was closed by a discussion of Nitrous Oxide, or Laughing Gas, and an interesting description of its generation in the liquid form and properties of the condensed gas by M. M. Johnston, of New York.

The subject of Mechanical Dentistry led to considerable dicussion on the merits of rubber and celluloid, there being quite a difference of opinion concerning the latter material.

Among the Voluntary Essays was an interesting paper on "The Dental Profession," by Prof. Jas. B. Hodgkin, of Washington City, D. C.

The Afternoon Session was devoted to Mechanical Dentistry, Therapeutics and Materia Medica.

The election of Officers for the ensuing year was then held with the following result:

*President.*—J. B. Patrick, Charleston, S. C.

1*st Vice President.*—L. D. Carpenter, Atlanta, Ga.

2*nd Vice President.*—V. E. Turner, Raleigh, N. C.

*3rd Vice President.*—J. B. Wood, Richmond, Va.

*Cor. Secretary.*—D. E. Everett, Raleigh, N. C.

*Rec. Secretary.*—E. S. Chisholm, Tuscaloosa, Ala.

*Treasurer.*—H. A. Lowrance, Athens, Ga.

*Executive Committee.*—W. H. Atkinson, New York; J. W. Selby, New York; S. J. Cobb, Nashville, Tenn.

New York was selected as the place, and the first Tuesday in September, 1880, as the time for holding the Twelfth Annual Session of the National Dental Association.

